# The angiotensin receptor blocker, Losartan, inhibits mammary tumor development and progression to invasive carcinoma

**DOI:** 10.18632/oncotarget.15553

**Published:** 2017-02-20

**Authors:** Rhiannon Coulson, Seng H. Liew, Angela A. Connelly, Nicholas S. Yee, Siddhartha Deb, Beena Kumar, Ana C. Vargas, Sandra A. O’Toole, Adam C. Parslow, Ashleigh Poh, Tracy Putoczki, Riley J. Morrow, Mariah Alorro, Kyren A. Lazarus, Evie F.W. Yeap, Kelly L. Walton, Craig A. Harrison, Natalie J. Hannan, Amee J. George, Colin D. Clyne, Matthias Ernst, Andrew M. Allen, Ashwini L. Chand

**Affiliations:** ^1^ Cancer Drug Discovery, Hudson’s Institute of Medical Research, Clayton, VIC, Australia; ^2^ Department of Anatomy and Developmental Biology, Monash University, Clayton, VIC, Australia; ^3^ Department of Physiology, University of Melbourne, VIC, Australia; ^4^ Cancer and Inflammation Laboratory, Olivia Newton-John Cancer Research Institute, Heidelberg, VIC, Australia; ^5^ Anatomical Pathology, Olivia Newton-John Cancer Research Institute, Heidelberg, VIC, Australia; ^6^ Anatomical Pathology, Monash Health, Clayton, VIC, Australia; ^7^ Department of Tissue Pathology and Diagnostic Oncology, Royal Prince Alfred Hospital, NSW, Australia; ^8^ Translational Breast Cancer Research, Garvan Institute, Darlinghurst, Sydney, NSW, Australia; ^9^ Sydney Medical School, Sydney University, NSW, Australia; ^10^ Tumor Targeting Laboratory, Olivia Newton-John Cancer Research Institute, Heidelberg, VIC, Australia; ^11^ Inflammation Division, WEHI, VIC, Australia; ^12^ Department of Pharmacology, University of Cambridge, Cambridge, UK; ^13^ Department of Physiology, Monash University, Clayton, VIC, Australia; ^14^ Translational Obstetrics Group, Department of Obstetrics and Gynaecology, University of Melbourne, Mercy Hospital, Heidelberg, VIC, Australia; ^15^ The ACRF Department of Cancer Biology and Therapeutics, John Curtin School of Medical Research, Australian National University, Canberra, ACT, Australia; ^16^ School of Cancer Medicine, La Trobe University, Heidelberg, VIC, Australia

**Keywords:** invasive ductal carcinoma, luminal breast cancer, angiotensin receptor, interleukin 6, tumor necrosis factor

## Abstract

Drugs that target the Renin-Angiotensin System (RAS) have recently come into focus for their potential utility as cancer treatments. The use of Angiotensin Receptor Blockers (ARBs) and Angiotensin-Converting Enzyme (ACE) Inhibitors (ACEIs) to manage hypertension in cancer patients is correlated with improved survival outcomes for renal, prostate, breast and small cell lung cancer. Previous studies demonstrate that the Angiotensin Receptor Type I (AT_1_R) is linked to breast cancer pathogenesis, with unbiased analysis of gene-expression studies identifying significant up-regulation of *AGTR1*, the gene encoding AT_1_R in ER^+ve^/HER2^−ve^ tumors correlating with poor prognosis. However, there is no evidence, so far, of the functional contribution of AT_1_R to breast tumorigenesis. We explored the potential therapeutic benefit of ARB in a carcinogen-induced mouse model of breast cancer and clarified the mechanisms associated with its success.

Mammary tumors were induced with 7,12-dimethylbenz[α]antracene (DMBA) and medroxyprogesterone acetate (MPA) in female wild type mice and the effects of the ARB, Losartan treatment assessed in a preventative setting (*n* = 15 per group). Tumor histopathology was characterised by immunohistochemistry, real-time qPCR to detect gene expression signatures, and tumor cytokine levels measured with quantitative bioplex assays. AT_1_R was detected with radiolabelled ligand binding assays in fresh frozen tumor samples.

We showed that therapeutic inhibition of AT_1_R, with Losartan, resulted in a significant reduction in tumor burden; and no mammary tumor incidence in 20% of animals. We observed a significant reduction in tumor progression from DCIS to invasive cancer with Losartan treatment. This was associated with reduced tumor cell proliferation and a significant reduction in IL-6, pSTAT3 and TNFα levels. Analysis of tumor immune cell infiltrates, however, demonstrated no significant differences in the recruitment of lymphocytes or tumour-associated macrophages in Losartan or vehicle-treated mammary tumors.

Analysis of AT_1_R expression with radiolabelled ligand binding assays in human breast cancer biopsies showed high AT_1_R levels in 30% of invasive ductal carcinomas analysed. Furthermore, analysis of the TCGA database identified that high AT_1_R expression to be associated with luminal breast cancer subtype.

Our *in vivo* data and analysis of human invasive ductal carcinoma samples identify the AT_1_R is a potential therapeutic target in breast cancer, with the availability of a range of well-tolerated inhibitors currently used in clinics. We describe a novel signalling pathway critical in breast tumorigenesis, that may provide new therapeutic avenues to complement current treatments.

## INTRODUCTION

Breast cancer is the most frequently diagnosed cancer in women, with an estimated 1.38 million new cases per year [1]. While breast cancer can be detected early and treated successfully, approximately 25% of patients become resistant to adjuvant treatments leading to advanced metastatic disease with poor prognosis. This highlights the necessity for new treatment options. One strategy, for identifying novel treatments, involves the repurposing of existing therapeutics. The observed influence of the blockade of the Renin-Angiotensin System (RAS) on cancer survival [2–5] combined with the known involvement of this system in inflammation suggests drugs targeting the RAS might be a promising therapeutic approach.

Expressed within different tissues, the RAS is a key endocrine pathway involved in the regulation of cardiovascular, renal and neuroendocrine function [6–9]. We now know that the RAS components, particularly the AT_1_R, are expressed in normal and tumor cell types including ovary, prostate, pancreas, breast and gut (reviewed by George *et al.* [10]). AT_1_R activation stimulates multiple signalling cascades important for the downstream control of angiogenesis, vascular remodelling, cell proliferation, differentiation, inflammation and fibrosis [9]. Given the importance of these processes in cancer, the inhibition of AT_1_R could offer a beneficial complementary treatment. In addition, as the RAS is expressed locally in tissues including the breast, it is most likely to be important for the regulation of “local” normal physiology in these organs. Further, aberrant RAS component expression may evoke tissue-specific processes of malignant transformation [10].

Angiotensin Receptor Blockers (ARBs) and Angiotensin-Converting Enzyme (ACE) Inhibitors (ACEIs) are now used successfully in the treatment of hypertension and other cardiovascular diseases. ARBs such as Losartan have no known off-target effects and are well tolerated in normotensive women. The use of ACEIs and ARBs to manage hypertension in cancer patients is correlated with improved survival outcomes for renal, prostate, breast and small cell lung cancer [2–5].

In 1998, a retrospective analysis demonstrated that among hypertensive medications, only ARB and ACEI users showed a decreased risk in lung and breast cancers [11]. This clinical finding was the first to suggest the RAS as being important in cancer progression. Subsequent retrospective analyses of datasets from randomised controlled trials have further supported this finding. Specifically assessing breast cancer cases, occurrence in ARB users (pre- and postmenopausal women) is 0.57% versus 0.85% in nonusers (*P* < 0.001; 42,921 subjects, in two matched groups for age, sex and comorbidities; ARB/ACEI exposure of ≥5 years) [12]. Use of ARBs and ACEIs also significantly decreased breast cancer recurrence [2].

The most conclusive evidence to date that links AT_1_R signalling to advanced breast cancer involved a Cancer Outlier Profile Analysis of gene expression profiling datasets from 3,157 microarray experiments [13]. One of the most consistently amplified gene was *AGTR1*, encoding the AT_1_R. *AGTR1* expression was up-regulated in 68% of the datasets analysed, attributed, in part, to changes in copy number [13]. *AGTR1* over-expression was observed to be approximately 100-fold higher in 10-20% of breast tumours, specifically in ER^+ve^/HER2^−ve^ primary tumours, positively correlating with poor prognosis and chemoresistance [13–15]. Elevated AT_1_R was also observed in a subset of metastatic tumor tissue [13]. While these studies shed some light on the association of AT_1_R with malignancy, the contribution of AT_1_R activity during malignancy still remains poorly understood.

## RESULTS

### Losartan treatment inhibits mammary tumour formation and progression *in vivo*

Given that postmenopausal women account for ~70% of all breast cancer cases, and are also more likely to be treated with ARBs for hypertension, we wanted to elucidate the effects of prophylactic ARB treatment on breast cancer development and progression *in vivo*. A chemical carcinogen (7,12-dimethylbenz[α]antracene (DMBA)) and medroxyprogesterone acetate (MPA) were used to induce mammary tumors in female wild type FVB/N mice (Supplementary Figure 1a). Tumors arising from combined DMBA and MPA treatment give rise to tumors that are both ERα positive, luminal tumours, as well as with basal-like characteristics [16–18]. We observed that tumours expressed ERα localised to the cytoplasm as well as nucleus, and expressed cytokeratin 8 suggesting a luminal subtype (Supplementary Figure 1f) as previously reported.

**Figure 1:**
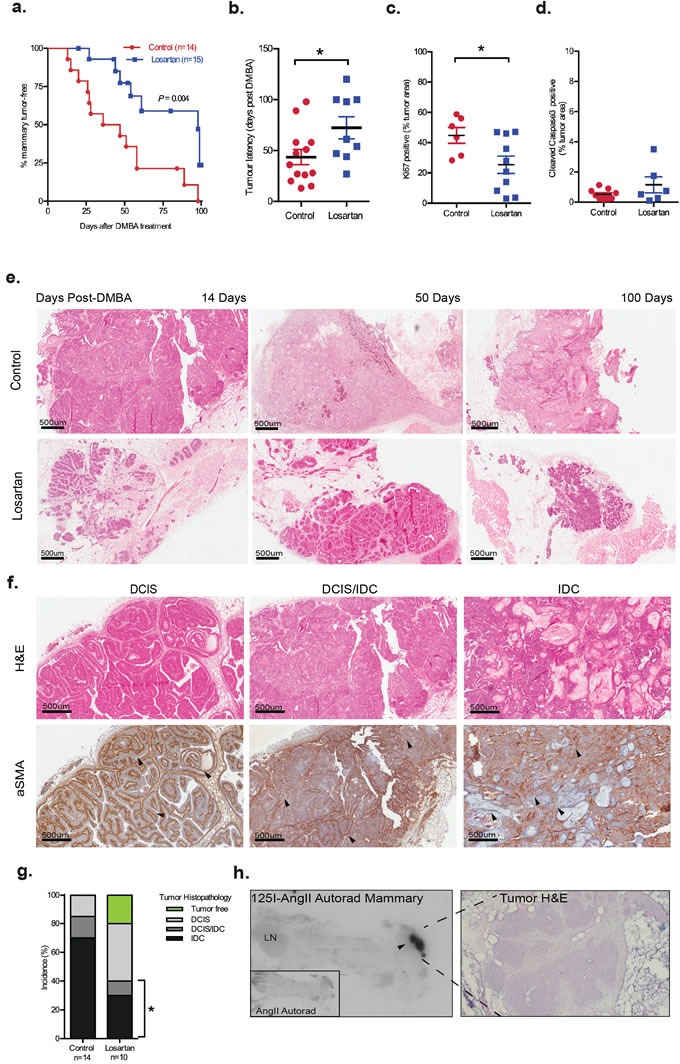
Inhibition of AT1R with Losartan decreases mammary tumor onset and progression associated with MPA and DMBA treatment Nulliparous six-week-old female mice were implanted subcutaneously with MPA pellets and treated orally with DMBA. Losartan (600 mg/L) was administered in drinking water. **a**. Onset of mammary tumors in age-matched wildtype MPA/DMBA-treated mice control-treated (3% sucrose) or with AT_1_R blocker, Losartan. Data for panels are expressed as percentage of mice free of tumors (palpable and histological assessment) after last DMBA treatment (*P* = 0.004, Mantel Cox Log-Rank Test). **b**. Tumor latency, development of palpable tumors days post-DMBA administration, data point represents individual animal. Immunohistochemistry quantification of **c**. Ki67 and **d**. Cleaved Caspase 3 in control and Losartan-treated animals. Each data point represents individual tumor analysed; and immunostain is presented as a percentage of total tumour area including tumour-associated stroma **e**. Representative Haematoxylin/Eosin (H&E) stained histological sections of tumors from age-matched control and Losartan treated animals. **f**. Representative histological sections of mammary tumor stages identified including Ductal Carcinoma *in situ* (DCIS), mixed DCIS and invasive ductal carcinoma (DCIS/IDC) and IDC; H&E stained (top panel) and immunostained with a differentiation marker for cancer-associated fibroblasts, α Smooth Muscle Actin antibody (αSMA, bottom panel). Intact myoepithelial layer identified by αSMA is indicated (arrowheads) in well-differentiated DCIS, while loss of myoepithelial αSMA and gain of αSMA expression in the stroma (arrowheads) is indicated in DCIS/IDC and the poorly differentiated squamous IDC. **g**. Tumor histopathology quantified as % of tumors assessed. Difference in incidence of invasive tumors between the 2 groups is indicated (*P* = 0.03, Fisher's Exact Test). Tumor-free animals are only observed in the Losartan group (green). **h**. Autoradiograph showing binding of I^125^-[Sar^1^, Ile^8^] Angiotensin II (I^125^AngII) to a section (unmagnified) of the inguinal mammary containing a DCIS lesion demonstrating strong AT_1_R expression. Insert: control tissue co-incubated with unlabeled AngII and I^125^AngII. H&E stained serial section of DCIS lesion at 20X magnification. Data are presented as mean +SEM, *n* = 10-15 animals per group. *P < 0.05, Mann Whitney Test unless otherwise stated.

Pharmacological blockade of AT_1_R signaling, with systemic Losartan treatment, significantly delayed mammary tumor onset (Figure 1a, *P* = 0.004 and Figure 1b, *P <* 0.05). Tumor cell proliferation, quantified by Ki67 immunostaining, was decreased in Losartan-treated animals (Figure 1c, *P* < 0.05), although no significant changes were observed in cleaved caspase 3 expression in tumors of Losartan-treated animals (Figure 1d, 0.5% in controls versus 1% in Losartan-treated). Strikingly, in the Losartan-treated cohort, 20% of animals (2 out of 10) were tumor-free and without cancerous lesions at the conclusion of the study, 100 days post DMBA treatment (Figure 1g). In contrast, none of the control animals were mammary tumor-free at the conclusion of the experiment (Figure 1f).

### Mammary tumours are histologically different in age-matched Losartan- and vehicle-treated animals

With the DMBA+MPA model, tumors display the histopathology of ductal carcinoma *in situ* (DCIS) and invasive ductal carcinoma (IDC) (Figure 1f). In DCIS, α smooth muscle actin (αSMA) was expressed distinctly in the myoepithelial layer, while in IDC, myoepithelial αSMA expression was lost, and differentiated cancer-associated fibroblasts (CAFs) acquired αSMA expression. Tumors of age-matched animals showed distinct histological differences between the two treatment groups (Figure 1e). The majority of tumors in control animals resembled high grade or metaplastic carcinoma with prominent squamous differentiation and/or a solid, papillary pattern (Figure 1e). In contrast to 85% of controls, only 40% of Losartan-treated animals developed IDC or had a mixed pathology of DCIS/IDC within their lesions (*P* = 0.03, Figure 1g). Progression to IDC was observed in 70% of tumors in the controls, compared to 30% of tumors in Losartan-treated mice (Figure 1g).

Previous characterisation of the RAS components including AT_1_R, using ligand-binding experiments, showed increased expression of AT_1_R within mammary tumors that developed in the MPA+DMBA model [19]. Utilizing the same radiolabelled AngII binding approach, we observed high AT_1_R levels in the tumor regions compared to the originating mammary gland (Figure 1h), suggesting that AT_1_R inhibition with Losartan can act locally within the tumor. The expression of AT_1_R mRNA in mammary glands did not alter with Losartan treatment (Supplementary Figure 1c). No changes in behaviour, feeding or body weight were observed in the animals in the two treatment arms (Supplementary Figure 1b), and no changes were observed in the overall mammary ductal morphology of age-matched control and Losartan-treated mice (Supplementary Figure 1d).

### AT1R-dependent cytokine production is a mechanism for mammary tumour progression

**Figure 2:**
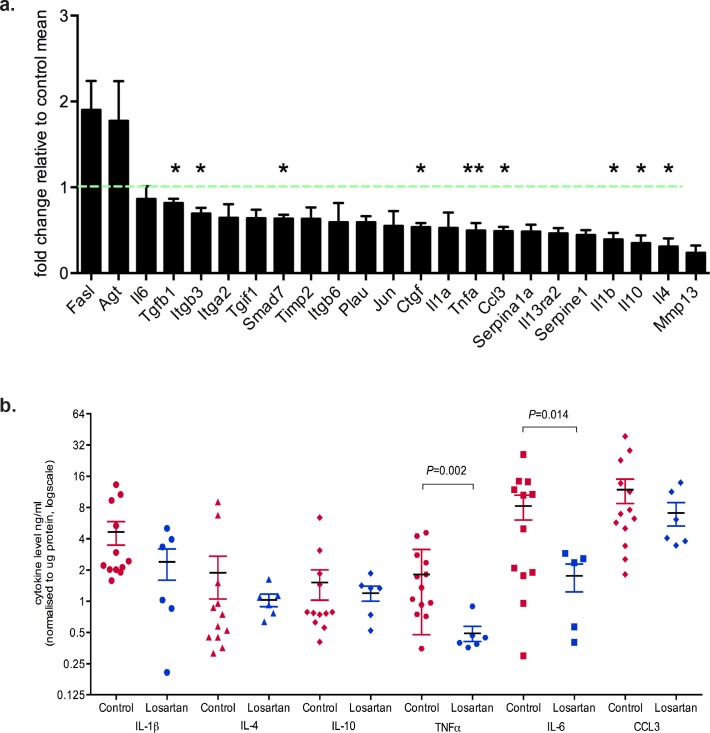

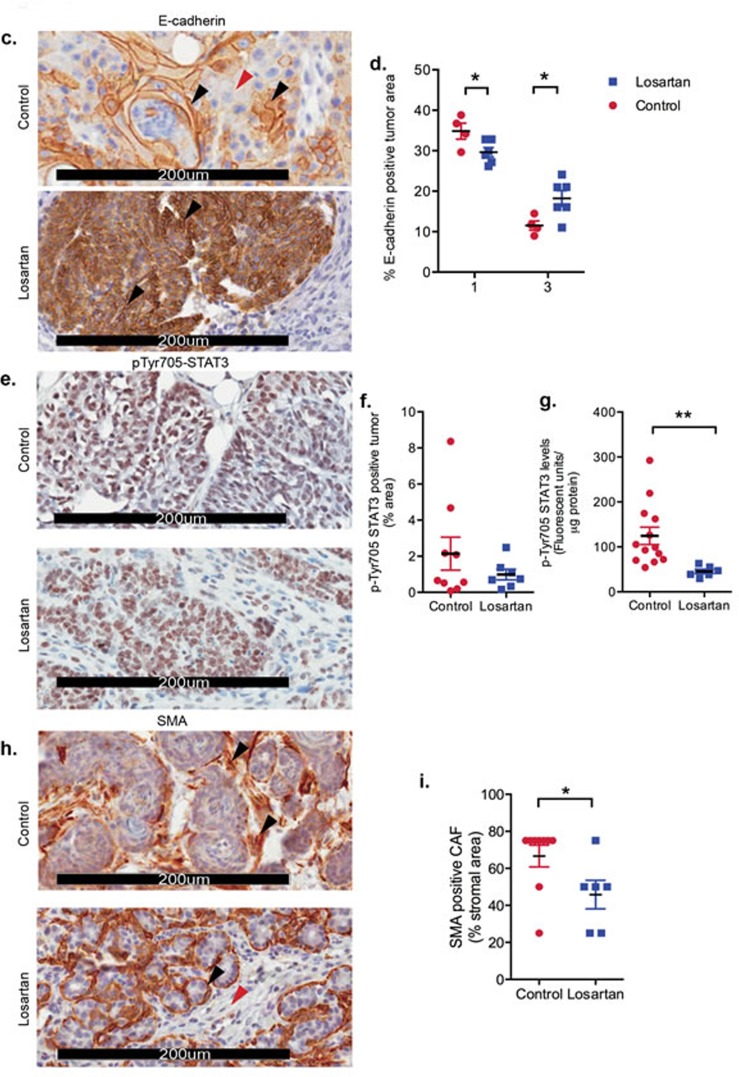
Inhibition of AT1R with Losartan decreases tumor cytokine production **a**. qRT-PCR expression of AT_1_R-regulated genes, with Losartan treatment, in mammary glands derived from mice 2 weeks after last DMBA-administration. Data is represented as fold change of mean gene expression in control samples (mean+SEM, *n* = 5 animals per group, unpaired Student's T-Test). **b**. Multiplex quantitation of cytokines in tumor lysates (mean+SEM, Control tumors n = 12 and Losartan-treated tumors *n* = 6). **c**. Representative immunostaining for E-cadherin, an EMT marker, in tumor sections from age-matched control and Losartan-treated animals. **d**. Quantitation of membrane-specific E-cadherin immunostaining intensity with ImageScope; score of 3 given to strongest stain (black arrows) and 1 given to weak/absence of stain (red arrows). **e**. Phospho-STAT3 localisation in age-matched tumors and the quantitation of pSTAT3 immunostain **f**. and with AlphaLISA in tumor protein lysates **g**. (mean+SEM, Control tumors *n* = 12 and Losartan-treated tumors *n* = 6). **h**. Representative α Smooth Muscle Actin (αSMA) immunostained sections from age-matched tumors highlighting greater proportion of αSMA positive CAFs in the tumor stroma in control animals compared to Losartan-treated tumors. Alteration in αSMA expression pattern in different tumor cell types is shown. In Losartan-treated tumors a strong myoepithelial staining in differentiated tumours (Losartan, black arrow) and the lack of αSMA in the CAFs (Losartan, red arrow). In control, vehicle treated animals CAF-specific αSMA expression is observed in the poorly differentiated, advanced tumors highlighted (Control, black arrows). **i**. Quantitation of αSMA positive cancer-associated fibroblasts (CAFs) presented as percentage αSMA-positive CAFs in the total tumor stroma (Control tumors *n* = 9 and Losartan-treated tumors *n* = 6). Representative tumor images are from serial sections taken from tumors 50 days after last DMBA administration. Data are presented as mean+SEM, *P < 0.05, *P < 0.01, Mann-Whitney Test unless otherwise stated.

To identify differentially regulated genes downstream of the AT_1_R in neoplastic mammary glands, we used RT2 Profiler Arrays in glands harvested two weeks after the last DMBA administration. We observed down-regulation of several cytokine transcripts in neoplastic mammary glands of Losartan-treated animals (Figure 2a). Significant decreases were measured for TGFβ1, integrin β3, connective tissue growth factor (CTGF), tumor necrosis factor α (TNFα), Macrophage Inflammatory Protein 1α (MIP-1α, CCL3), interleukin (IL) 1, IL-4 and IL-10 mRNA (Figure 2a), all of which are implicated in inflammation, fibrosis and wound healing processes.

Next we determined whether the AT_1_R-dependent changes in cytokine mRNA transcripts were reflected at the protein level within the tumors. Using multiplex ELISA, we observed a significant reduction in tumor cytokine levels of TNF (*P* = 0.002), and IL-6 (*P* = 0.014) in Losartan-treated compared to control tumors (Figure 2b). IL-6 is a potent inflammatory cytokine involved in tumor inflammation, epithelial to mesenchymal transition (EMT) and tumor progression. Elevated IL-6 serum levels directly correlate with disease staging and poorer clinical outcomes in women with metastatic breast cancer [20].

Therefore, we assessed E-cadherin expression as a marker for EMT [21] (Figure 2c). Strong membrane E-cadherin expression was observed in tumors from Losartan-treated mice compared to control mice (Figure 2c-2d, *P* < 0.05), suggesting a potential IL-6-mediated inhibition of tumor de-differentiation following ARB treatment. Strong evidence of a functional link between inflammation and breast cancer cell transformation has been shown to be mediated by the activation of NF-κB signalling, increased IL-6 and STAT3 phosphorylation by Iliopoulos and collegues [22]. We measured tumor phospho-Tyr705-STAT3 to determine whether the decrease in IL-6 levels resulted in the suppression of STAT3 activation. Nuclear pSTAT3, which localised to tumor epithelial cells and stroma in control mice (Figure 2e), showed reduced expression in Losartan-treated tumors, reflective of the decrease in IL-6 levels (Figure 2f). Additional validation by SureFire AlphaLISA also showed a reduction in pSTAT3 levels (Figure 2g, *P* < 0.05).

Within the breast tumor, the highest levels of IL-6 are found on the tumor edge, in stromal/immune cells and areas of lymphovascular invasion [23]. Differentiation of fibroblasts within and adjacent to the tumor, referred to as cancer-associated fibroblasts (CAFs), is a key driver of tumor growth and provides a primary source of estrogen [24–26], promoting tumor cell invasiveness [27] and metastatic dissemination. Tumor epithelial cell-derived cytokines, including IL-6 and TNFα, induce CAF activation or desmoplasia [27, 28], characterised by SMA expression [29]. In comparison to controls, mammary tumors from Losartan-treated animals show reduced αSMA-positive CAFs (Figure 2g-2h) indicating reduced CAF activation. Prophylactic treatment with Losartan resulted in an anti-inflammatory response mediated by reduced IL-6 and TNFα production, which prevented the differentiation of tumor stroma and epithelial cell EMT, and attenuated the malignant transformation of DCIS to IDC. Our data implicates a therapeutic window in which prophylactic ARB use may be efficacious in controlling cancer cell dedifferentiation and the acquisition of an invasive phenotype.

### Losartan treatment affects tumor vasculature and not infiltration of immune cells

**Figure 3:**
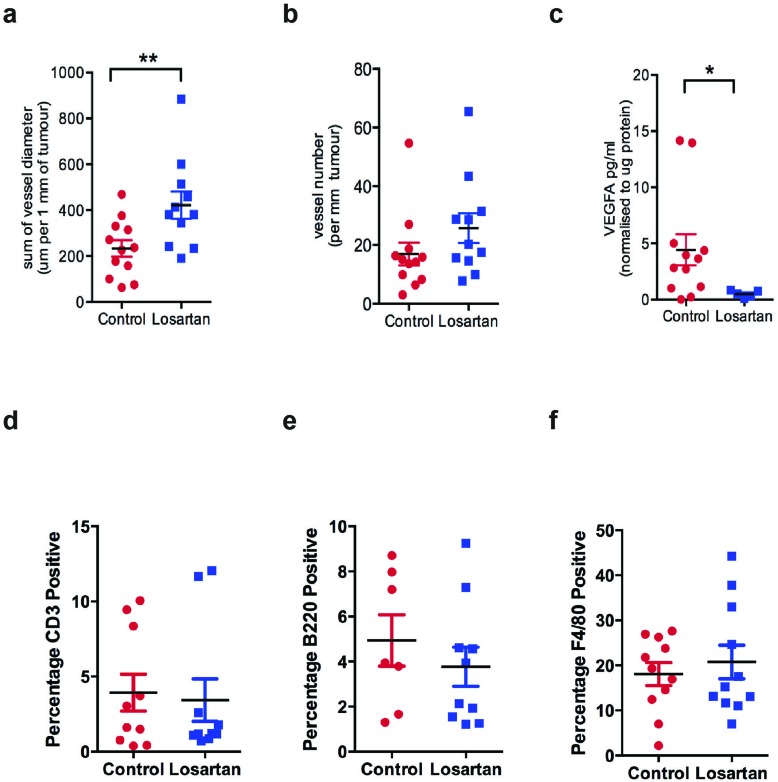
Losartan treatment increases peripheral blood vessel diameter and reduces VEGFA levels in tumors **a**. Vessel diameter was measured for the blood vessels located at the tumor circumference, **b**. blood vessel density and **c**. VEGFA, a marker for angiogenesis, measured by ELISA in tumor lysates derived from control and Losartan-treated animals. Quantitation of immunohistochemistry stain of **d**. CD3, a marker for T cells, **e**. B220, a marker of B cells and **f**. the macrophage marker F4/80, in mammary tumour tissue of the two treatment groups. Data are presented as percentage of positively stained areas of the total tumour area including tumour-associated stromal regions; mean+SEM, *P* < 0.05, Mann Whitney Test.

ARBs are widely prescribed and by far the most successful treatment available for heart disease and hypertension. Interestingly, Chauhan *et. al*. recently demonstrated that Losartan treatment improved the delivery of chemotherapeutic agents into pancreatic and breast cancer xenografts via the reduction in tumor solid stress and the decompression of tumor blood vessels [30]. We observed that Losartan treatment caused a significant increase in blood vessel diameter measured at the periphery of the tumor (Figure 3a, *P* < 0.01), without altering the number of blood vessels (Figure 3b). Given the prominent effects of angiotensin II on vascular growth, we quantified tumor expression of VEGFA by ELISA, and found VEGFA levels were significantly decreased in tumors of Losartan-treated animals (Figure 3c, *P* < 0.05) and a possible downstream effect of reduced IL-6 levels in response to AT_1_R inhibition [31].

Given that we observed a reduction in the levels of proinflammatory cytokines IL-6, IL-1β and TNFα within the tumour, we investigated changes in immune cell populations within the tumour and its microenvironment. We performed immunohistochemistry with markers for T lymphocytes (CD3+, Figure 3d), B lymphocytes (B220/CD45R+, Figure 3e) and tumour-associated macrophages (F4/80+, Figure 3f). There were no differences in cells positive for CD3, B220 or F480 in *Losartan* versus untreated mammary tumours, reflecting no changes in the recruitment of lymphocytes or tumour-associated macrophages (TAMs).

### AT1R expression is elevated in luminal breast tumors

**Figure 4:**
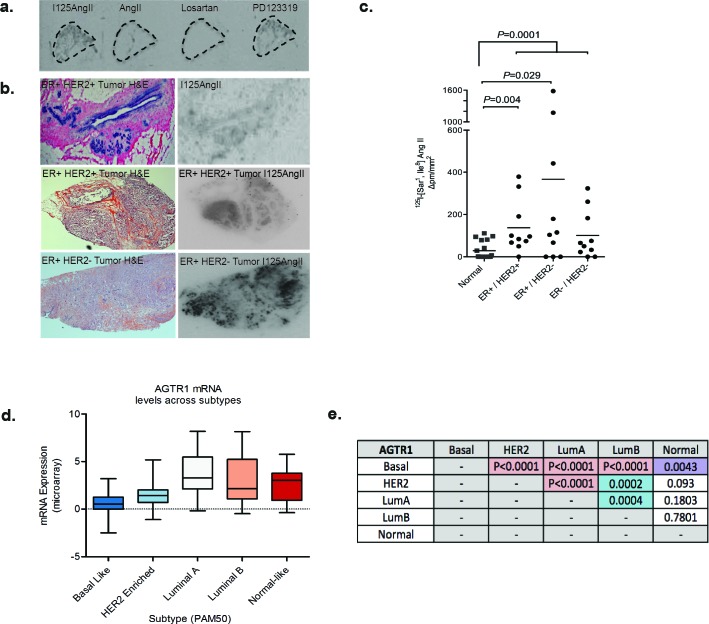
Expression of AT1R is increased in invasive breast cancer **a**. Binding of ^125^I-[Sar^1^, Ile^8^] AngII to serial sections of a Grade II, ER^+ve^ invasive tumor. Radioligand binding is displaced by unlabelled AngII or Losartan (AT_1_R blocker), but not PD123319 (AT_2_R blocker) indicating expression of AT_1_R. **b**. *Left-hand panel*: H&E-stained breast carcinoma sections; *Right-hand panel*: Corresponding ^125^I-[Sar^1^, Ile^8^] AngII binding in serial sections. **c**. Mean intensity of bound ^125^I-[Sar^18^] AngII /total area in tumor subtypes and matched normal tissue, *n* = 10; data points are combined from matched normal for each sub-type, *n* = 30. **d**. *AGTR1* mRNA expression in TCGA breast invasive carcinoma, subtyped by the PAM50 molecular signature and **e**. *P* values highlighting statistically significant differences in *AGTR1* expression between the PAM50 tumor subtypes (Mann Whitney Test). TCGA data was retrieved from cBioPortal.

The use of ARBs as a potential breast cancer therapy would be predicted to have maximal success in patients with tumors expressing AT_1_R. As the *AGTR1* gene is amplified in ER^+ve^/HER2^−ve^ Grade II and III breast tumors [13], we assessed AT_1_R expression in human breast carcinoma samples. Due to the lack of specific antibodies, AT_1_R levels in matched normal and invasive carcinoma samples were quantified with a competitive ligand-binding assay. Frozen tumor tissue sections were probed with ^125^I-[Sar1, Ile8]AngII, and co-incubated with unlabeled AngII, Losartan or PD123,319 (an inhibitor for AT_2_R, a functionally distinct isoform of AT_1_R). Competitive inhibition of binding was observed with unlabeled AngII and Losartan (Figure 4a), indicating detectable AT_1_R, but not AT_2_R, in tumor and normal tissue.

The AngII binding density was highest in the epithelial compartment of normal breast and tumor tissue sections. Furthermore, AT_1_R levels are significantly higher in the tumor regions than adjacent normal tissue (*P* = 0.0001, Figure 4c), as also observed in our *in vivo* tumor model (Figure 1h). Compared to normal breast, high AT_1_R expression occurs in 73% of tumors analyzed. The mean AT_1_R density was significantly elevated in ER^+ve^/HER2^+ve^ (5-fold, *P* < 0.004) and ER^+ve^/HER2^−ve^ (13-fold, *P* < 0.03) IDC, relative to the mean AT_1_R levels in matched-normal breast (Figure 4c). Analysis of the TCGA (The Cancer Genome Atlas) dataset, using the prediction analysis of microarray 50 (PAM50) subtype gene signature as a classifier, reveals significantly higher mean *AGTR1* expression in Luminal A tumors compared to Luminal B, HER2 overexpressing and basal subtypes (Figure 4d and Figure 4f). In agreement with our ligand-binding data, *AGTR1* mRNA expression in the TCGA dataset shows an inverse correlation to HER2 expression across the PAM50 subtypes (Figure 4d).

Longitudinal studies of patients diagnosed with DCIS reveal that 20-50% will go on to develop IDC [32–35]. Tumor heterogeneity of Luminal breast cancer, particularly Luminal A arising from different molecular profiles, accounts for the varied responses to current treatments and the inability to accurately predict the potential for recurrent and metastatic disease. We show that breast tumors highly express AT_1_R and propose that these tumors would be responsive to ARB treatment. This proposal is based on our findings in the DMBA+MPA *in vivo* model, where systemic treatment with Losartan results in a significant delay in the progression of DCIS lesions to invasive and de-differentiated tumors, through the down-regulation of IL-6-dependent STAT3-phosphoryation.

## DISCUSSION

In the present study, we demonstrate that the inhibition of AT_1_R, with Losartan, in a hormonally driven, chemically induced model of breast cancer results in a significant delay in tumour onset. In 20% of Losartan treated animals, 20% remained free of mammary tumors. This data suggests the importance of AT_1_R driven signalling in the development of mammary tumours. We also observed a significant difference in the histological characteristics of tumours between vehicle controls and Losartan-treated animals. In Losartan-treated animals, majority of tumors resembled DCIS lesions while age-matched control tumours were histologically akin to invasive tumors. Additionally a reduction in tumour cell proliferation, measured by Ki67 staining was observed in the tumours from Losartan-treated animals. The differences in tumour progression observed in age-matched animals may in part be explained by a reduction in tumor IL-6, pSTAT3 and TNFα levels; suggesting that the tumour cytokines play a role in AT_1_R-mediated tumorigenic functions. Recent reports have shown elevated *AGTR1* mRNA levels in breast cancer tissue [13] and our radiolabelled-ligand binding data is supportive of this observation. In this study we show that when compared to normal breast tissue, high AT_1_R expression is found in 73% of tumors, with >10-fold higher AT_1_R levels observed in 30% of tumors. Our analysis of the TCGA database is confirmatory that high *AGTR1* expression is associated with luminal breast cancer and inversely correlated to HER2 expression.

The renin-angiotensin system is a key endocrine pathway involved in the regulation of cardiovascular and neuroendocrine function [6–8]. Locally, within different tissues the renin-angiotensin system has diverse paracrine functions associated with cellular fibrosis, proliferation and differentiation [9]. This system comprises of angiotensinogen, secreted by the liver, cleaved to AngI in the circulation by an enzyme, renin. The ACE then modifies AngI into AngII in the lungs.

AngII is the classical main effector endocrine hormone that acts via two G-coupled protein membrane receptors: type 1 (AT_1_R), and a functionally distinct type 2 (AT_2_R). AT_1_R-dependent actions including angiogenesis, vascular remodelling, proliferation, inflammation and fibrosis are well characterized in cardiovascular tissue. Thus, components of the RAS represent a critical pharmacological target in the treatment of cardiovascular disorders with the development of ACEI and ARBs. These are now used successfully for the treatment of hypertension and other cardiovascular diseases.

Within the breast, the RAS components have been localised to epithelial ducts of normal breast and tumors [36, 37]. Another study demonstrates that the RAS components, angiotensinogen, renin, ACE and AT_1_R are expressed in hormone-receptor negative and ER^+ve^ human breast cancer tissue, as well as in representative human breast cancer cells [38]. Interestingly, angiotensin II-mediated stimulation significantly increased VEGFA gene expression only in ER^−ve^ cell lines, which was completely inhibited by the ARB, Candesartan [38]. A recently published study also confirms a pro-tumourigenic role of AT_1_R demonstrating that overexpression of AT_1_R in an ER^+ve^ breast cancer cell line resulted in increased proliferation, increased expression of poly(ADP-ribose) polymerase (PARP) and X-linked inhibitor of apoptosis (XIAP), increased ERK, p-Smad3/4 activation [39]. Furthermore, treatment with Losartan attenuated these effects. Overexpression of AT_1_R also accelerated tumor growth, increased tumor angiogenesis, enhanced tumor invasiveness as demonstrated by increased expression of EMT markers including matrix metallopeptidase 9 (MMP-9) and reduced E-cadherin [39]. Collectively these studies identify the mechanisms via which AT_1_R facilitates tumor growth and invasiveness in breast cancer.

The importance of AT_1_R function on mammary gland morphogenesis during normal development and pregnancy has been characterised by Nahmod and colleagues using mice deficient in either, or both, of the two different murine angiotensin receptors (AT_1A_ or AT_1B_) and with the administration of Irbesartan, an ARB [40]. Of the two angiotensin receptors, AT_1A,_ the ortholog of human AT_1_R, mediated signaling was important in tissue remodeling during postlactational mammary gland involution. Furthermore, systemic and local administration of AngII into lactating female mice induced phosphorylation and nuclear translocation of STAT3, which was inhibited by Irbesartan, suggesting that the activation of these signalling pathways was indeed mediated by AT_1_R [40]. In our mammary carcinogenesis model, we also observed a significant reduction in phosphoSTAT3 in tumors of Losartan-treated mice.

Furthermore, we demonstrate that the inhibition of tumor progression from DCIS to IDC is mediated by the suppression of local production of the inflammatory cytokines IL-6 and TNFα, within the tumor and mammary tissue. AT_1_R inhibition has proven to reduce inflammation in hypertension, as well as LPS-induced inflammation, via the reduction of IL-1β, IL-6 and TNFα levels [41]. In addition, treatment with BAY-117082 (NF-κB inhibitor) and a monoclonal antibody against IL-6 significantly suppress tumor growth in xenografts of breast, prostate and colon cancer cells [22], indicating the importance of these pathways in tumor cell transformation. IL-6 is known to alter the phenotypes of various cell types within the tumor microenvironment to secrete chemokines and VEGF [22, 31], enhance vasculature permeability and angiogenesis, and promote tumor cell dissemination and metastasis [23].

Adaptive and innate immune responses are important in tumor immunosurveillance and tumour infiltrating lymphocytes (TIL) in breast cancers correlate to better responses to chemotherapy and disease prognosis. Within the mammary tumor and surrounding stromal areas, we did not observe alterations in TILs (T or B lymphocytes) in samples from the two treatment arms, Macrophages play an important role during the neoplastic transformation of mammary epithelial cells, including the facilitation of DNA damage via reactive oxygen release, enhancing cell survival and creating a chronically inflammatory state within the tumour via secretion of the proinflammatory cytokines TNFα, IL-6 and IL-1β [42]. Given the decrease observed in the levels of above-mentioned cytokines in Losartan-treated tumours, we assessed the accumulation of TAMs but did not observe any significant changes in TAM numbers in the tumour or associated-stroma. Further investigation into the mechanisms via which AT_1_R and IL-6 drive tumour epithelial cell de-differentiation and effects on immune cells including the cytotoxic T cell function and macrophage-polarisation status are warranted.

Recent data indicate that ARBs and ACEIs have beneficial effects in reducing tumor progression, vascularisation and metastasis. Candesartan, an ARB, reduced tumor angiogenesis and lung metastases in a mouse lung cancer model [43]. In an *in vivo* model of colorectal cancer liver metastases, both Captopril (an ACEI) and Irbesartan (an ARB) significantly reduced tumour growth, angiogenesis and percentage of liver metastases [44]. These responses to Candesartan treatment were also observed in xenograft models of human prostate [45] and ovarian cancer [46]. Reduction in tumor growth rates in human pancreatic, breast and prostate cancer cell xenografts are observed with treatment of the ARB, Losartan [13, 30, 47]. Losartan treatment also improved the delivery of chemotherapeutic agents into pancreatic and breast cancer xenografts via reduction in solid stress and the decompression of tumour vessels [30].

There is also evidence for the effects of the RAS in the tumor stroma, highlighted in experiments where a significant reduction in tumor growth is observed when melanoma cells are transplanted into AT_1_R-knockout mice [48]. Further to this, a large body of evidence suggests that AT_1_R activation could regulate tumor cell secretion of growth factors and cytokines into the tumor microenvironment, enhancing the growth of fibroblasts and vascular-forming endothelial cells and subsequent tumor cell proliferation [10].

The overexpression of components of the RAS, in cancers including brain, lung, pancreatic, breast, prostate, colon, skin and cervical cancers relative to their corresponding non-malignant tissue implicate important local actions within the tumor. In our study, we show increased AT_1_R abundance in human invasive breast carcinoma biopsies, and in DMBA+MPA induced mouse mammary tumors; and that the systemic treatment with Losartan is effective in reducing tumor burden in mice. The mammary epithelial and stromal proliferative index is highest during the luteal phase of the menstrual cycle, or in women receiving combined estrogen and progesterone hormone replacement therapy (HRT). Significant increases in breast density, breast cancer risk and recurrence are also observed during pregnancy, and when progestins are included in HRT. Hence the mammary tumour model utilising a synthetic progesterone (MPA) and a chemical carcinogen (DMBA) has provided a relevant model to specifically determine the effects of AT_1_R in the mammary gland undergoing remodelling involved in pre-neoplastic hyperplasia and tumor initiation. Studies involving the generation of a double transgenic *agtr1−/−*/MMTV-PyVT model, and the use of syngeneic metastatic breast cancer lines in the *agtr1−/−* host would be beneficial in further investigating the therapeutic importance of the RAS in the development of mammary tumor development and in metastatic disease. With such models the effects of AT_1_R in tumour epithelial and stromal fibroblast and immune cells could be clearly delineated.

In conclusion, our findings indicate a specific role of AT_1_R inhibition in delaying the occurrence and progression of invasive breast cancer. It is proposed that the inhibition of the AT_1_R, and its down-stream signaling through IL-6, could provide a promising treatment avenue for breast cancer, utilizing a well-characterized and tolerated class of ARB/ACEI drugs. Our data suggests that ARB treatments could complement treatments targeted at inducing cytotoxicity by reducing tumor inflammation and cellular transformation of tumor epithelial and stromal cells.

## MATERIALS AND METHODS

### Study approval

All animal experiments were performed in accordance with the ethical requirements of the Monash University Animal Ethics Committee (Ethics Approval # MMCB2012/29). For studies undertaken utilizing human breast tissue and tumor biopsies, experiments were performed with ethics approval from the Monash Health Ethics Committee (12105B).

### MPA and DMBA induction of mammary tumors *in vivo*

The MPA and DMBA model of mammary tumor induction was used as previously described [18]. In brief, single 50mg, 90-day release medroxyprogesterone acetate (MPA) pellets (Innovative Research of America, Sarasota, FL) were subcutaneously implanted under general anaesthesia in 5-8-week old female wild-type FVB/n mice. Following recovery, animals were randomly divided into either treatment or control groups and provided with either 600mg/L Losartan with 2% w/v sucrose *ad libitum* in drinking water as previously published [47, 49, 50], or 2% w/v sucrose water, respectively, for the remainder of the study. The daily intake of water is ~3ml, equating to ~70mg/kg/day. In mice, 70mg/kg/day is routinely used for *in vivo* expts without any adverse effects on the general health of the animals including changes in behavior, water intake, alterations to blood pressure. We did not observe any changes in body weight (Supplementary Figure 1).

After 10-16 days of Losartan treatment, 4 weekly doses of the chemical carcinogen 7,12-dimethylbenz(a)anthracene (DMBA) at 1mg in 0.1 mL sunflower oil was administered by oral gavage, with a one week break between the second and third dose (Supplementary Figure 1a). Two experimental time points were assessed, 14 days after DMBA treatment or up to 100 days after (*n* = 15 per treatment group for each experiment). Power calculations with a 95% confidence interval determined that *n* = 15 per treatment group is a sufficient number of animals to provide statistically significant findings. In the long-term study, animals were monitored for palpable mammary tumour development three times per week and weighed twice weekly. Animals were euthanized upon detection of palpable mammary and/or other tumours >5mm in diameter, a loss of more than 10% body weight over a period of a week, or other observed signs of illness or distress. The MPA pellet was replaced after 90 days during the experiment duration of 100 days. Animals in the short-term study were used to investigate Losartan action on early tumour initiation mechanisms active in the mammary gland.

### Mammary tissue collection and analysis

The fourth inguinal mammary gland from each animal was fixed in 4% w/v PFA. The opposite inguinal (ninth) mammary gland was whole mounted for gross mammary gland morphological analysis as previously described [51]. Tumors or visible dysplasia were harvested and fixed in PFA and snap frozen for subsequent analysis. Remaining tumor free glands were also collected and snap frozen.

Morphological assessment of mammary gland whole-mounts entailed the quantification of ductal branching and lobulo-alveolar (LA) gland growth. The entire gland was imaged sequentially at 4x magnification and the mammary gland development in each field scored based on a devised rating scale (Supplementary Figure 1d). An average LA score from all fields was plotted for each animal. Tumour histology was annotated by two breast cancer pathologists, who performed blind assessments independently, of the H&E stained tumour sections. αSMA staining (AbCam, ab5694, 1:200) was used to differentiate benign hyperplasia, *in situ* tumours and invasive carcinoma. Proportion of dysplasia in non-tumour containing mammary was also examined.

### Gene expression analysis

Quantitative reverse-transcriptase PCR (qRT-PCR) was performed on RNA isolated from tumor and whole mammary lysates. Briefly, total RNA was prepared using Trizol^®^ LS Reagent (Life Technologies, Carlsbad, CA) as per manufacturer's instructions, treated with DNaseI (DNA-free™ DNA Removal Kit, Life Technologies), and quantified using a NanoDrop 1000 Spectrophotometer. First strand cDNA synthesis using 1.0 μg total RNA was performed using SuperScript^®^ III First Strand Kit (Life Technologies) primed by random hexamers. mRNA was quantified using TaqMan probes (AT1aR: Mm01957722_s1, RN18S: Mm03928990_g1) and Taqman Gene Expression Master Mix (Life Technologies) on the ABI Prism 7900-HT Real-time PCR system. Fold change in expression was calculated using the comparative CT method (ddCt) method after normalisation to 18S as the internal control.

Pathway expression analysis was performed using Applied Biosystems’ (Life Technologies) RT2 Profiler PCR Arrays for 84 genes involved in human breast cancer (PAMM-131ZA) and fibrosis (PAMM-120ZA) according to the manufacturers protocol. 400ng input RNA isolated from whole mammary tissue lysates was reverse transcribed to cDNA using the RT2 First Strand Kit. qRT-PCR was then performed using RT2 SYBR Green ROXTM qPCR Mastermix on the ABI Prism 7900-HT Real-time PCR system in 384 well plates. Samples from 5 animals per group, harvested 2 weeks post DMBA treatment. Gene expression was quantified using the ddCt by normalizing to 5 housekeeping genes, and expressed as fold-change relative to the control.

### Immunohistochemistry

Sections (5 μm thickness) from PFA-fixed, paraffin-embedded mammary glands and tumor tissue were de-waxed and rehydrated in graded ethanol washes. Antigen retrieval was performed on the sections immersed in citrate buffer (pH 6.0) and heated in a 1000W microwave at 100% power for 3 min, 30% power for 7 min, and left to cool for 20 min. Sections were then treated with 3% H_2_O_2_ for 5-10 min to block endogenous peroxidase activity. Non-specific binding was blocked using 5% v/v serum (source of secondary antibody) for 1 hour at room temp. Tissues were then incubated overnight at 4°C with the primary antibody of interest including: αSMA (Abcam ab5694, 1:200), Ki67 (Abcam ab833, 1:400 and Thermoscientific (SP6), 1:200), E-cadherin (BD610182, 1:300), F480 (in-house Ludwig antibody, 1:200, trypsin antigen retrieval), Cleaved Caspase 3 (Cell Signalling #2215, 1:300), phosphoSTAT3 (Cell Signalling #2215, 1:200) and ERα (Santa Cruz sc-542, 1:200). Vectorstain Elite ABC Kit for Rabbit IgG, (PK-6101, Vector Laboratories) was utilized for detection according to the manufacturers instructions.

Antigen-antibody complexes were detected by staining with 3, 3′-diaminobenzidine tetrahydrochloride (DAB) Liquid Substrate Kit (Dako) and sections counterstained with haematoxylin (Sigma Diagnostics). Stained sections were imaged with an Aperio Slide Scanner. Whole tumor sections were selected and immunostaining for E-cadherin, Ki67, pSTAT3 and cleaved caspase 3 were quantified using Aperio ImageScope Positive Pixel Count Algorithm, written for the detection of nuclear, cytoplasmic or membrane staining. The algorithm measurements included signal intensity and calculation of the fraction of pixels with a positive-color measurement. 2 independent investigators also assessed staining patterns. Data is presented as percentage positive pixels of the total number of pixels counted. Immunostaining was measured as a percentage of the total tumour area including epithelial and stromal cells. Three investigators independently made blind assessments of αSMA immunostaining in cancer-associated stroma. Data is presented as mean + SEM, with each data point representative of a tumour from a separate animal.

### Cytokine quantitation with multiplex ELISA

Levels of IL-1β, IL-4, IL-6, IL-10, TNFα and MIP-1α were measured using quantitative Milliplex Luminex (MilliPlex MAP Mouse Cytokine Panel 8-plex, Millipore, Australia) assays according to the manufacturer's instructions. Standards and tumor protein lysates (25μl) were incubated with antibody coated beads overnight with mild agitation at 4°C. Detection antibodies were incubated for 1 hour at room temperature and the fluorescent conjugate Streptavidin-Phycoerythrin was added to each well and incubated for 30 min at room temperature (RT). Analysis of each sample was performed in duplicate and the enzyme-linked immunosorbent assay (ELISA) run on 2 separate occasions. Positive and negative quality controls are included on each assay in duplicates. The lower detection limit was 3.2 pg/ml for all analytes, while the intra-assay variability was less than 10%. Data were collected and analysed using a BioPlex 200 instrument equipped with BioManager analysis software (Bio-Rad). Standard curves for analytes generated with Analyst 5.1 using 5-parameter log curve. Samples were corrected for total μg protein added to assay wells. Each data point is representative of a tumour from a separate animal.

### AlphaLISA SureFire p-STAT3(Tyr705) assay

pSTAT3 levels were quantified in tumor protein lysates using AlphaLISA *SureFire Ultra* pSTAT3 (Tyr705) Assay Kit (PerkinElmer, Cat # ALSU-PST3-A500) according to manufacturer's instructions. The assay entailed incubation of 5 μl of tumor lysate with CaptSure donor and acceptor beads coated with antibodies against the phospho-Tyr705-epitope and the distal STAT3α and STAT3β (NP_644805 and NP_998827). In the presence of pSTAT3, the proximity of the two antibodies induced a fluorescent signal measured by the EnSight Plate reader. Samples were corrected for total μg protein added to assay wells and each sample assayed in triplicate. Each data point is representative of a tumour from a separate animal.

### Quantitative autoradiography

Clinical tumour biopsy samples of invasive ductal carcinoma, pathological stage 2 or 3, and matched normal mammary tissue, were obtained from Victorian Cancer BioBank and assessed for AT_1_R expression with radioligand binding assays as previously described [52]. Clinical parameters of patients are reported. 20 μm frozen sections of tumor and normal breast tissue sections were incubated with 0.2 pCi/ml ^125^I[Sar1, Ile8] Angiotensin II (AngII) (90 pmol/L, ProSearch, Australia) to determine total receptor binding, 10 mmol/L PD123319 (AT_2_R isotype blocker) to show AT_1_R specific binding, and/or Losartan to show AT_1_R binding or 1 mmol/L unlabelled Ang II (Sigma, Australia) to show nonspecific binding. Additionally, mouse mammary tissue containing tumour lesions were also assessed for AT_1_R expression with ^125^I[Sar1, Ile8] Angiotensin II radioligand binding.

Slides were washed and dried after incubation and exposed to X-ray film at room temperature for 14 days. In each cassette, a set of ^125^I-radioactivity standards were included and optical density values calculated from the standard curve generated. The developed film was scanned using a microcomputer image analysis system and Scion Image Software (Scion Corp, Frederick, MD, USA) used to measure signal intensity. This approach was undertaken as most commercially available antibodies tend to have non-specific activity. The binding of [^125^I]AngII was calculated by extrapolation of signal intensity values of autoradiographs with known concentrations of [^125^I]AngII in an 8 point standard curve, as previously published [53].

### Assessment of tumor vasculature

A trained pathologist made blind assessment of tumor vascularization using the Aperio ImageScope software. Measurements of total tumor area, tumor circumference, average vessel diameter and total of blood vessels present at tumor periphery were recorded. Vessel number and total sum of vessel diameter values were normalised to tumor area assessed.

### Statistical analysis

Data was analyzed using PRISM software for statistical analysis with Student's T-Test, Mann-Whitney U test (if data were not distributed normally), Mantel Cox Log-Rank Test and Fisher's Exact Test used accordingly. *P* < 0.05 was considered significant.

## SUPPLEMENTARY MATERIALS FIGURE



## Abbreviations

AngII: Angiotensin II
AT1R: Angiotensin Receptor Type I
AGTR1: AT_1_R gene name
ACE: Angiotensin-Converting Enzyme
ACEI: Angiotensin-Converting Enzyme Inhibitors
ARB: Angiotensin Receptor Blockers
αSMA: alpha Smooth muscle actin
CAF: cancer-associated fibroblasts
CTGF: connective tissue growth factor
CCL3/ MIP-1α: Macrophage Inflammatory Protein 1α
DCIS: Ductal carcinoma in situ
DMBA: 7,12-dimethylbenz[α]antracene
ER+ve: Estrogen Receptor positive breast cancer
HER2-ve: Herceptin receptor negative breast cancer
HRT: Hormone replacement therapy
IDC: Invasive ductal carcinoma
IL: Interleukins 1β, IL-4, IL-10, IL-6
MPA: Medroxyprogesterone acetate
pSTAT3: phosphorylated Signal transducer and activator of transcription 3
RAS: Renin-Angiotensin System (RAS)
TNFα: tumor necrosis factor alpha
VEGFA: Vascular endothelial factor A
