# Supplementary material for: The angiotensin receptor blocker, Losartan, inhibits mammary tumor development and progression to invasive carcinoma
Source: Oncotarget. 2017 Feb 20;8(12):18640–56. doi: 10.18632/oncotarget.15553 (PMC5386636; doi:10.18632/oncotarget.15553)
Supplement: Supplementary file 1 [file oncotarget-08-18640-s001.pdf]

The angiotensin receptor blocker, Losartan, inhibits mammary tumor development and progression to invasive carcinoma

Supplementary Material

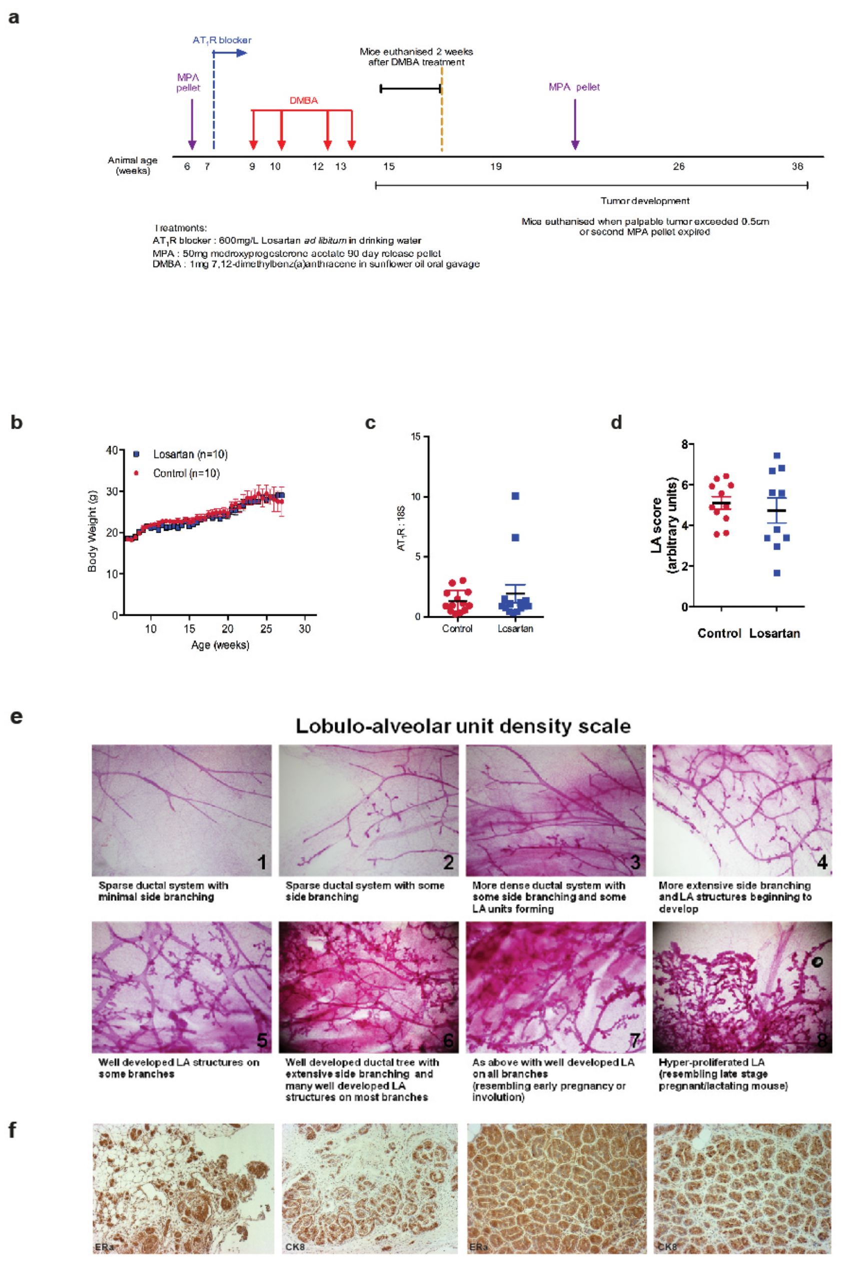

Supplementary Figure 1: Carcinogenesis model for mammary tumor induction and ductal morphological assessments. (a) Timeline depicting schedule for Losartan, medroxyprogesterone (MPA) and dimethylbenzanthracene (DMBA) treatments. (b) Animal body weights over treatment course. (c) AGTR1 mRNA expression in mammary glands lysates. (d) Assessment of mammary gland development by quantitation of lobulo-alveolar structures. (e) Assessment criteria for mammary gland development in carmine alum stained mammary gland whole. (f) Representative immunohistochemical stained sections for Estrogen Receptor α (strongly and weakly positive) and cytokeratin 8 as luminal markers.
